# Incorporating reading strategies for EFL undergraduate learners in Saudi Arabia: A think-aloud study

**DOI:** 10.12688/f1000research.123094.1

**Published:** 2022-09-20

**Authors:** Arif Ahmed Mohammed Hassan Al-Ahdal, Yasamiyan Alolaywi

**Affiliations:** 1 Department of English Language and Translation, College of Sciences and Arts, Methnab, Qassim University, 51931, Saudi Arabia

**Keywords:** EFL Learners, Reading Strategies, Hearing Abilities, Language Instructors

## Abstract

Background: In language learning, reading is a skill that enables interaction with a text in whatever field of knowledge the learner is pursuing. Readers tend to use strategies such as comprehension, interpretation and conception of decoding written language and texts to enhance their reading capacity. This research explores the reading strategies applied by Saudi English as a Foreign Language (EFL) learners and compares the reading abilities of male and female EFL students. Methods: The study interviewed three EFL students about the reading strategies they applied while reading passages and texts. Then an International English Language Testing System (IELTS) reading test was handed over to 26 randomly selected students. Results: The study found that the reading strategies used by the students interviewed involved skimming, scanning, guessing the meaning from context, identifying the mean idea, and summarizing the body of the text in question. Moreover, the study revealed that both male and female students scored low in the IELTS tests that the researchers conducted. The average mean score they reached was 7.15 out of 20. However, even in the low scoring ratio, female students (M=7.69) outperformed male students (M= 6.62) and the difference between them remained significant, P=.000. Conclusions: The study recommends that the language instructors help EFL learners in developing their reading strategies and applying them every time they read any text.

## Introduction

While talking and hearing abilities are gained from infancy or through time, reading is actually a learned skill that can be strengthened. Since reading involves intellectual functions like encoding additional knowledge, using cognitive skills, engaging a layout for current understanding; it is different from literacy. The skills used in literacy, on the other hand, invite various strategies and metacognitive and cognitive approaches that define learners’ awareness of which method works best for them and then selecting them. Substantial research and triarchic theory of intelligence by Sternberg points out that students with high achievement rates seem to be more metacognitive than students with lower achievement rates ( Sternberg, 1985). Although simply having that metacognitive skill is not enough; factors such as motivation, persistence, focus and self-reliance play an important role for a successful path to learning.

Language researchers have shown a keen interest in the ‘think-aloud’ (expressing the thoughts as they occur) technique as a reading strategy. The technique provides ample data for further research into cognitive processes and this study used the same technique with the undergraduate EFL learners in the classrooms of Saudi Universities. Despite the studies that have been conducted previously, there are limited studies that identify the reading levels of the Saudi EFL learners and their IELTS performance levels. Also, there has been no investigation of the reading strategies used by EFL learners and how they impact them across gender divides. This paper attempts to explain how the undergraduate male and female participants approach the reading strategies. It also denotes how a ‘think-aloud’ approach can be viewed through a qualitative as well as quantitative lens where the participants are more like quasi-researchers who voice the words in their heads while trying to complete the assigned tasks.

## Literature review

Reading strategies have been discussed widely in previous research ( Li, 2020). Reading strategies play a major role in helping students to understand texts ( Suraprajit, 2019; Al-Ahdal, 2020). However, tertiary level students were found to have decisive problems in reading academic texts because of their use of ineffective reading techniques ( Al-Mekhlafi, 2018). Kuhn (2000) defines metacognition as what enhances metacognitive awareness of what one thinks and how one recognizes and maintains strategic control while applying these strategies to process new information. Some of these strategies have been used over a period of time by the students to help them in comprehension. It has been suggested that ‘think-aloud’ (expressing the thoughts as they occur) is a particularly effective strategy for readers who struggle while reading an unfamiliar text ( Olshavsky, 1997).

The studies of reading strategies spread over many dimensions ( Al-Mekhlafi, 2018; Bagci & Unveren, 2020; Deliany & Cahyono, 2020; Deliany & Cahyono, 2020; Suraprajit, 2019; Wahyono, 2019; Al-Ahdal & Al-Awaid, 2014). Wahyono (2019) explored the correlations between reading strategies and reading comprehension while Deliany and Cahyono (2020) investigated EFL students’ use of metacognitive reading strategies. Similarly, Suraprajit (2019) detected the use of bottom-up and top-down reading strategies in reading academic business language. Al-Mekhlafi (2018) also investigated the EFL learners’ frequent use of reading strategies. Reading approaches, as according to Fandiño *et al.,* (2019), may be divided into three categories: global schemes, dilemma (e.g., rereading), and supporting reading skills (e.g., taking notes).

### Reading comprehension strategies

Much research has been undertaken in the journals on various elements of strategy usage and metacognitive strategies in reading ( Dąbrowska, 2018; Li & Clariana, 2019; Zhang *et al.*, 2019; Guo *et al.*, 2018) by emphasizing the significance of instructional tactics in literature classrooms (Ferrara *et al.*, 2020). Ample research concentrating on the effects of instructional techniques on students’ reading skills show that activities have a favorable impact on the EFL learners ( Altiok *et al.*, 2019; Jin *et al.*, 2020). Biological sex and competency variations on techniques usage have also been identified in related studies (e.g., Dąbrowska, 2018; Guo *et al.*, 2018; Li & Clariana, 2019; Jansen *et al.,* 2019). Variability between good readers and those who have difficulty, according to Dąbrowska (2018), are influential determinants in the adoption of effective learning strategies. Furthermore, Jansen *et al.* (2019) performed a survey in a scenario where a lack of competence in the goal of linguistic knowledge (English) was asserted, and the investigator found that competency was indeed a determinant in adopting techniques for learning. Likewise, research conducted by Guo *et al.,* (2018) studied how Chinese EFL students observe self-regulatory strategies in reading. Results showed that fluency was a predictor among the EFL students who incorporated several methods in their reading approach. Garner’s (1990) theory of settings proposes that strategies need to be changed according to the context. The classroom instructions do not always support the strategy used and the learners fall back on their old routine practices instead of adapting to the new methods.

*Think-aloud methods as reading techniques*

The think-aloud strategy for reading has been adapted by several researchers ( Bai, 2018; Camilo *et al.,* 2020; Follmer & Sperling, 2018; Huang, 2019; Lan, 2020; Li & Clariana, 2019; Reiber-Kuijpers *et al.,* 2021; Wei, 2020). They employed think-aloud procedures in their studies for the process of recognizing metacognitive strategies. Li and Clariana (2019) conducted think-aloud research with 26 students of various levels of proficiency and they found that proficiency was beneficial in the application of several techniques. Along the same lines, Reiber-Kuijpers *et al.* (2021) employed think-aloud techniques to compare the strategies that both the successful readers and the less advanced readers used while reading and found that successful readers used many more tactics. Similarly, utilizing think-aloud protocols, Lee *et al.* (2019) determined several learning techniques in the outcomes of a study including 80 learners. The study revealed that developing literacy strategy and teaching reading skills should be tackled in a comparable research area. Tinzmann *et al.* (1990) ascertain that even training students to the thinking aloud habit keeps the classroom interactions robust. Ozek and Civelek (2006) consider the think-aloud strategy as an effective assessment tool to explore students’ reading strengths and weaknesses as it gives an insight to the reader’s mind which is otherwise unobservable ( Block, 1986). Gunning (1996) asserts that think-aloud can be used to comprehend features such as seeking information using prior knowledge, visualizing texts, observing and solving word or comprehension related problems. The role of reflection and guidance from the teacher cannot be ruled out, if the teacher needs the reading strategy to work and for that they need to provide an environment where the student can think while they are reading ( Chamot and O’Malley, 1994, p.11 cited by Lavadenz, 2003; Farr & Conner, 2004).

### Reading strategies

Reading strategies have been the focus of many previous studies ( Al-Mekhlafi, 2018; Bagci & Unveren, 2020; Deliany & Cahyono, 2020; Deliany & Cahyono, 2020; Suraprajit, 2019; Wahyono, 2019; Al-Ahdal & Almarshedi). For example, Deliany and Cahyono (2020) studied EFL students’ implementation of metacognitive reading tactics and reported that most students had high metacognitive reading strategies. Also, the study found that there was no significant difference between the male learners and the female learners in the use of reading techniques. Furthermore, Suraprajit (2019) examined the use of bottom-up and top-down reading strategies in academic business language in a study that used a questionnaire to collect data from Thai students. The study reported that the top-down strategy was the most commonly used method of instruction. It focused on activities that constructed meaning by utilizing background knowledge, making predictions and searching the reading material to assert or discard the predictions that were made. In contrast, the bottom-up strategy focuses on the text which is read and analyzed from start to finish. Students did want to use their time reading aloud, identifying tenses or dividing the sentences into small elements. This was the least preferred method of instruction.

Moreover, Al-Mekhlafi (2018) investigated strategies commonly used by the EFL learners. The study found that students used different reading strategies extensively and there was no significant difference between the levels of students in the use of reading strategies. Similarly, Nazurty *et al.*, (2019) studied how frequently the Indonesian student teachers use reading strategies. The study investigated the use of reading strategies at pre-reading level, reading level and post-reading level. The findings showed that cognitive reading strategies were used by EFL students in all three different phases of reading. Furthermore, the study reported the tendency of female students to use cognitive strategies while male students used metacognitive strategies. Additionally, Wahyono (2019) explored students’ perceptions on reading strategies and their scores in reading comprehension in studies that indicate the correlation between reading strategies and comprehension of students. Findings of the study showed that the four cognitive pre-reading strategies including preview, prediction, prior knowledge, and purpose were used by all the students. In addition to those, many students used cognitive reading strategies such as rehearsal, developing new language, summarization, guessing meaning from context, and employing imagery for memorization. Furthermore, the study reported a correlation between reading strategies and reading comprehensions with a coefficient of 0.61. Think-Aloud Protocols (TAPs) provide valuable information about how learners solve problems, what obstacles they face, how and in what contexts they use these strategies in their learning tasks ( Someren *et al.*, 1994).

### Research questions

Using the Think-aloud-protocol pattern, this study aims to answer the following questions:

1. What reading strategies do EFL students employ when answering reading questions?
2. Are there any significant differences in the level of reading ability between the male students and the female students?

## Methods

### Study design

A mixed-method research approach was utilized in the study as data were analyzed both quantitatively and qualitatively. A reading test was used to assess students’ ability and applicability of the reading strategies. This is quantitatively assessed. Furthermore, the qualitative data were collected by interviewing three students during the reading session. The data collected through the two instruments were mixed in answering the research questions to triangulate and validate the findings. This study employed a think-aloud protocol design to gather verbal data on cognitive and metacognitive reading strategies used by undergraduate students enrolled in the EFL department in Qassim University for the academic year (1443AH). This is an explanatory design relying on the verbal protocols of the participants. Data analysis aimed to explore both cognitive and metacognitive reading strategy types that the participants used while engaged in a reading task.

### Study population

The study sample consisted of 26 EFL students selected from the Department of English and Translation, College of Sciences and Arts, Methnab, Qassim University that were divided equally by gender (13 males and 13 females). All the students were native speakers of Arabic majoring in English. The ages of the participants ranged from 20 to 22 years. These students were randomly assigned to the study from a group of students who were enrolled in a Reading and Vocabulary Building Course. The selection of participants was based on the criteria of using the odd numbers from the list of students. Afterwards, three students (two males, and one female) were purposefully selected based on their proficiency levels to participate in the reading thinking-aloud task and to answer the interview questions. Table 1 below shows some demographic data about the study sample:

**Table 1.** Learners' demographic characteristics.

Number of Participants	26
Age Range	20-22
Specialization	English Language & Translation
Level	Junior (Sophomores)
Gender	13 Males & 13 Females

### Data collection

***Instruments***

**Reading test**

A reading test was administered to the participating EFL students to measure their reading abilities and to engage them in think-aloud sessions. The reading was selected from an already valid and reliable IELTS reading test taken from ( Hayes & Read, 2004) and can be found under *Extended data* (a copy of the interview questions can be found under *Extended data* (Al-Ahdal Arif, ‘Incorporating reading strategies’, 2022). IELTS testing is an international standardized testing of English language proficiency for non-native English language speakers with an established worldwide credibility to assess language fluency. The test consists of a reading passage from a general topic and a set of twenty questions where each question is worth one point (Appendix A). The questions have different formats involving multiple-choice questions, matching, and summarizing the text.

**Interviews**

The three purposefully selected students underwent a semi-structured interview session in order to explore their perceptions and prior knowledge of reading strategies. The interviews were a part of the think-aloud sessions which were conducted with an effort to gain more in-depth information about the respondents’ reading habits. The interview questions were adapted from Ghavamin, *et al.* (2013) (a copy of the interview questions can be found under *Extended data* (Al-Ahdal Arif, ‘Incorporating reading strategies’, 2022). The interview focused on the learning and reading strategies that the learners used while reading a text and the steps they take to improve upon their reading abilities.

***Think-aloud sessions***

**Procedure**

Initially, twenty-six undergraduate students majoring in English were assigned to the current study (13 males and 13 females). The students were required to complete a reading comprehension test during a Reading and Vocabulary Building class in one hour. Afterwards, three participants were purposefully selected to take part in a think-aloud session to verbalize all the thoughts and processes going on in their minds while they were taking the test. As stated above, the interview questions were adapted from Ghavamin, *et al.* (2013). This means that the questions were already valid and reliable in the original study. Furthermore, the researchers administrated them on two students who were excluded from the study. The students reported the clarity and understandability of the interview questions. The researchers recruited them based on their previous verbal consent to participate in the study.

Every week, the instructor explained to the students a new reading strategy (e.g., scanning, skimming, previewing, guessing the unknown word, etc.) Students in each think-aloud session were required to apply the studied reading strategies to the target reading texts. They were also asked to report what they had comprehended from the text aloud. This procedure continued for the whole semester and at the end of the semester, the impact of the think-aloud strategy was assessed through a reading test. Students also reported their knowledge about the strategies they mastered and applied through an interview.

The think-aloud protocols were then recorded using a mobile phone and their responses transcribed by the researchers after. The interview was held in the English Department with three students. It took 15 minutes for each student to answer the five interview questions. As stated earlier, the interview was held in English, and when there was a language barrier, Arabic was used. Furthermore, the answers were transcribed, cleaned and themed. They appear in the results section along with the students’ number, i.e. (student 1-3). Then, the data were decoded and classified into categories based on the reading strategies adopted by these sampled students. The types of reading strategies adopted by the EFL learners while performing the reading task were identified and examined. The researchers also analyzed the extent to which gender differences played a role in learners’ comprehension and utilization of the reading strategies learned over a course of time.

**Ethics statement**

Ethical approval was obtained from the Scientific and Ethical Committee of Qassim University on (29/3/2022). Informed verbal consent was obtained from participants for the use and publication of their data.

**Data analysis**

Data for this research were gathered during the second semester of the academic year 2021-2022. Data were analyzed both quantitatively and qualitatively. The raw scores obtained from the reading tests were coded and calculated using statistical tool SPSS or Statistical Package for the Social Sciences, (Version 26) used for complex statistical data analysis. Descriptive statistics were reported involving minimum and maximum scores, frequencies, mean scores along with the standard deviation. Moreover, data gathered from the semi-structured interviews during the think-aloud sessions were analyzed qualitatively. This was done manually by applying the following steps: The students’ answers were anonymously presented using the word ‘Student’ to maintain the privacy of those participants. As soon as the data were transcribed, they were cleaned; a close examination of the students’ transcripts was required to identify appropriate themes and classifications. Each student’s responses were divided into five parts to answer the five interview questions. In this study, the analysis was conducted at sentence level. This means that every student’s interview was semi-open so, they were transcribed into short sentences focusing on the reading strategies they used (see the quotations of students 1 to 3 below). Wherever appropriate, quotes from the students’ responses were also used to support the themes identified in the tests.

## Results

*Research Question 1. What reading strategies do ESL students employ when answering reading questions?* This question is answered through analyzing the students’ responses at the interview stage of the study. Students used various reading strategies depending on the types of reading questions they answered. They reported using the following reading strategies: reading for overall understanding, scanning and guessing the meaning, identifying the part of speech of certain vocabulary, and summarizing. The full results can be found under *Underlying data* (Al-Ahdal Arif, ‘Incorporating reading strategies’, 2022) *.* Their answers were: “In the beginning, I read mainly to get the gist of the text. For further reading, I scan the text and try to guess the meaning from the body of the text. It helps me identify the part of speech being used in the text and I’m able to guess the meaning from the context of the passage. Using this information, I summarize the main idea that emerged from the text,” (Student 1). Similar strategies were also reported by the previous student: “I use all three types of strategies: skimming, scanning and guessing the meaning of the text from the context of the passage,” (Student 2). Furthermore, the same focus was mentioned by another student: “I use guessing contextual meaning from the text drawing inferences, identifying part of speech and comprehending what the writer intends to say,” (Student 3).

*RQ2. Are there any significant differences in the level of reading ability among male and female students?* The second research question will be answered using the students’ responses to the IELTS test. According to Table 2, the average mean score of the students was 7.15 and the standard deviation was 3.173. Male students’ mean score in the reading test was (M=6.62) with a standard deviation of (Std=2.663) whereas the mean score of female students was (M=7.69) and standard deviation of (Std=3.683) in the reading test. Female students outperformed male students in the reading competence and the difference in their scores was significant. The *P* value is.000 which is less than.05. We found there was a significant difference in reading ability between the male and female students in the study. Female students used reading strategies more effectively than their male counterparts during the reading tasks. They applied reading strategies, i.e., skimming, scanning, guessing the meaning of unfamiliar words using the context, etc. more frequently and effectively.

**Table 2.** Students’ results in the reading test.

Sex	Mean	Std. Deviation	T	df	Sig. (2-tailed)
Male	6.62	2.663	7.531	12	.000
Female	7.69	3.683	8.958		
Average	7.15	3.173			

## Discussion

The present research intends to investigate the reading strategies used by EFL students in order to get insight into their cognitive abilities. The study reported that students used various reading strategies depending on the reading questions. Some of the strategies were: reading to get an understanding of the main point of the text, skimming, scanning, guessing the meaning from the context, identifying the central idea and summarizing it. Students’ use of such reading strategies enabled them to comprehend various types of texts which developed their cognitive abilities used for language learning and acquisition. Many studies found that EFL learners used reading strategies to comprehend the academic reading texts as well. The study proved that the students employed cognitive and meta cognitive reading techniques while reading and that they preferred a top-down approach to a bottom-up approach. Michael Yeldham (2004) cites Vandergrift in his study of top-down and bottom-up approach who agrees that strategy instruction needs to be predominantly top-down, because then the learners become more aware using the information they already have to fill gaps in their understanding ( Vandergrift, 2004).

The students used reading strategies extensively and their level of learning didn’t factor highly into using these strategies. While the study of the Indonesian student teacher reading programs showed that the EFL learners used different learning strategies during pre-reading and post-reading periods, their reading abilities improved with the wide variety of learning strategies used over a period of time. The study by Hoang Nguyen and Daniel R. Terry (2017) of Vietnamese EFL learners and staff found that the English learning success was attributed to multiple elements and not any single factor therefore the use of strategies and teachers’ flexibility in adapting the strategy to the needs of their students go hand in hand.

The students did not fare so well in the IELTS reading test with a mean score of 7.15 out of 20. Male students scored (6.62) lower than female students (7.69) and the difference between them is significant. In this, the finding differed from Deliany and Cahyono (2020), who reported that there was no difference between male and female EFL students using metacognitive reading strategies. This finding may co-relate the findings of Nazurty *et al.* (2019) who reported that while the female students used more cognitive strategies, the male students used metacognitive strategies. In a study to train the students’ metacognitive skills based on gender differences, Nunaki, Damopolii, Kandowangko, and Nusantri (2019) developed a 4-D research model to test both male and female students of a senior high school in Indonesia. The study supports the findings that there are no differences in the metacognitive skills used by the male or female students in inquiry-based learning.

## Conclusions

The global scheme, rereading and supporting reading skills methods serve a variety of functions, but they all stem from complicated cognitive processes. In this research, scanning techniques, questionnaire/interviewing and testing of the participants were used. These studies used think-aloud protocols to assess EFL’s students’ metacognition and usage of worldwide reading skills while taking into account regularly used approaches in developing language studies. The study also suggests that students need to be trained to develop their metacognitive skills and that strategies alone do not result in successful outcomes. With the combined efforts of language instructors, curriculum design experts and motivated students, a successful implementation of strategies and effective learning can take place. As a result, the impact of instructional strategies could be demonstrated in aiding the reading abilities of the students. The study seeks to assess how EFL students approach and utilize think-aloud protocols in light of the relevant research and earlier investigations. Think-aloud is both a method of inquiry and a flexible mode of goal-oriented instruction. The success of its implementation depends on how well it can be used in classroom teaching. Some well-rehearsed exchanges between the teacher and the students are required to judge the competence levels of their EFL learners. Thus, incorporated into instructional approaches in schools in Saudi Arabia, think-aloud can lead to a constructive and engaging classroom environment that facilitates textual comprehension as well as social interaction.

### Recommendations for further studies on reading strategies in the Saudi context:

1. EFL instructors need to give a combination of prompts that are relatable and culturally familiar.
2. Instructors need to refrain from giving any feedback during the reading process even if students mispronounce or misread certain words. Only when the student has read their piece should the researchers interfere.
3. The instructors can introduce simple warm-up exercises before they begin using the think aloud technique to make the task easier for the learners.
4. Switching back and forth from the native language to the foreign language might complicate the learning process and confuse the EFL learners so the instructor needs to ensure that only the target language is used in the process.
5. Non-verbal cues such as body language and gestures and paralinguistic features such as tone of the voice and rhythm of the speech along with verbal expressions play an important role in learning a foreign language and instructors need to take note of that and include it in their data collection.
6. Pairing up students can be an interesting feature of further studies where one student reads and the other one listens and takes notes and vice versa.
7. Instructors are advised to extend the activities to other features of language learning such as short story building, storytelling and poetry reading activities in groups.
8. Instructors need to provide a follow-up to their study so that the students recognize their efforts and work on their limitations.
9. Instructors’ consistent use of learning strategies for academic purposes will motivate language learners to follow suit.
10. Instructors can model the activity by ‘role-playing’ or practicing the strategy with the learners before they incorporate it into their lesson plans.
11. Further research exploring the gender differences based on age, grade-level and competency level can provide new insight on the success of cognitive-led studies.

### Limitations

1. The study did not investigate the metacognitive reading techniques that can be further used in similar studies.
2. If prompts are too complicated and require interpretation, they can be counter-productive to the learning intended for the participant.
3. While the participant is trying to read by thinking aloud, questions should not be asked during the process as it may break their rhythm of thought process.
4. Time restrictions may shorten the scope of the activity planned.
5. Verbalization alone may not be an effective approach.
6. Breaking into native language patterns might impede the progress made during the learning activity.

## Data availability

### Underlying data

Figshare: Incorporating reading strategies for EFL undergraduate learners in Saudi Arabia: A think-aloud study. https://doi.org/10.6084/m9.figshare.20401917.v1. ( Al-Ahdal Arif, ‘Incorporating reading strategies’, 2022)

This project contains the following underlying data:

- Raw Scores.xlsx
- Results.docx
- Transcriptions of the students Responses.docx
- Students’ paper.pdf
- Dataset.sav

### Extended data

This project contains the following extended data:

- Interview Questions.docx
- Reading Test.docx

Data are available under the terms of the Creative Commons Attribution 4.0 International license (CC-BY 4.0).
