# Supplementary material for: Incorporating reading strategies for EFL undergraduate learners in Saudi Arabia: A think-aloud study
Source: F1000Res. 2022 Nov 8;11:1070. Originally published 2022 Sep 20. [Version 2] doi: 10.12688/f1000research.123094.2 (PMC9647143; doi:10.12688/f1000research.123094.2)
Supplement: Peer review history

# Peer review: Reviewer response for version 2

**DOI:** 10.5256/f1000research.139269.r155233

**Published:** 2022-11-10

**Authors:** Asmara Shafqat

**Affiliations:** 1 Department of English, Humanities, NED University of Engineering and Technology, Karachi, Pakistan

The authors have addressed all the issues and revised the article as per the concerns raised. It is now approved, with no further revisions required.

Is the work clearly and accurately presented and does it cite the current literature?

Partly

If applicable, is the statistical analysis and its interpretation appropriate?

Partly

Are all the source data underlying the results available to ensure full reproducibility?

Yes

Is the study design appropriate and is the work technically sound?

Yes

Are the conclusions drawn adequately supported by the results?

Yes

Are sufficient details of methods and analysis provided to allow replication by others?

Yes

Reviewer Expertise:

Academic reading and writing, Corpus linguistics, Pragmatics, Discourse, Corpus Driven Teaching Methods, Learning styles of the learners and teachers. EAP and ESP and all ELT issues.

I confirm that I have read this submission and believe that I have an appropriate level of expertise to confirm that it is of an acceptable scientific standard.

# Peer review: Reviewer response for version 2

**DOI:** 10.5256/f1000research.139269.r155234

**Published:** 2022-11-09

**Authors:** Ahdi Hassan

**Affiliations:** 1 Global Institute for Research Education & Scholarship, Amsterdam, The Netherlands

I have gone through the revised version of the article and I find it in a perfect shape. In its current form, it would be of great significance to the teachers and learners of English in an EFL setting. I am quite certain it would also be a very valuable reference for researchers to fall back on.

Is the work clearly and accurately presented and does it cite the current literature?

Yes

If applicable, is the statistical analysis and its interpretation appropriate?

Yes

Are all the source data underlying the results available to ensure full reproducibility?

Yes

Is the study design appropriate and is the work technically sound?

Yes

Are the conclusions drawn adequately supported by the results?

Yes

Are sufficient details of methods and analysis provided to allow replication by others?

Yes

Reviewer Expertise:

Applied Linguistics, corpus linguistics, English language

I confirm that I have read this submission and believe that I have an appropriate level of expertise to confirm that it is of an acceptable scientific standard.

# Peer review: Reviewer response for version 2

**DOI:** 10.5256/f1000research.139269.r155232

**Published:** 2022-11-09

**Authors:** Gilbert C. Magulod jr

**Affiliations:** 1 College of Teacher Education, Cagayan State University, Tuguegarao, Philippines

The revised paper has an acceptable quality to be indexed and read by the community of practice. The paper has academic and managerial significance to the community.

Is the work clearly and accurately presented and does it cite the current literature?

Yes

If applicable, is the statistical analysis and its interpretation appropriate?

Partly

Are all the source data underlying the results available to ensure full reproducibility?

Yes

Is the study design appropriate and is the work technically sound?

Yes

Are the conclusions drawn adequately supported by the results?

Yes

Are sufficient details of methods and analysis provided to allow replication by others?

Partly

Reviewer Expertise:

NA

I confirm that I have read this submission and believe that I have an appropriate level of expertise to confirm that it is of an acceptable scientific standard.

# Peer review: Reviewer response for version 1

**DOI:** 10.5256/f1000research.135163.r150896

**Published:** 2022-09-27

**Authors:** Gilbert C. Magulod jr

**Affiliations:** 1 College of Teacher Education, Cagayan State University, Tuguegarao, Philippines

The paper can contribute to the EFL undergraduate learners in Saudi Arabia. The study is a mixed methods research which is commendable. However, the following should be considered to revise the paper: 

1. In the introduction, there is a need for the author to present the problem in the global issue down to the local issue. 
2. In the abstract, the qualitative finding should be emphasized. it should also reflect a separate discussion of the qualitative component. What themes have been developed? 
3. The author should provide another statement of the problem focusing on the qualitative component? 
4. For the quantitative component, present the normality of data to justify the use of inferential statistics. 

The paper can be a contributory to enhance reading strategies of EFL learners.

Is the work clearly and accurately presented and does it cite the current literature?

Yes

If applicable, is the statistical analysis and its interpretation appropriate?

Partly

Are all the source data underlying the results available to ensure full reproducibility?

Yes

Is the study design appropriate and is the work technically sound?

Yes

Are the conclusions drawn adequately supported by the results?

Yes

Are sufficient details of methods and analysis provided to allow replication by others?

Partly

Reviewer Expertise:

NA

I confirm that I have read this submission and believe that I have an appropriate level of expertise to confirm that it is of an acceptable scientific standard, however I have significant reservations, as outlined above.

# Peer review: Reviewer response for version 1

**DOI:** 10.5256/f1000research.135163.r150898

**Published:** 2022-09-27

**Authors:** Ahdi Hassan

**Affiliations:** 1 Global Institute for Research Education & Scholarship, Amsterdam, The Netherlands

Incorporating reading strategies for EFL undergraduate learners in Saudi Arabia: A think-aloud study: Review Report

1. The paper is well written and organized. It lays down the reading strategies as applied by Saudi EFL students along with the "think aloud" variable. The abstract gives a clear and concise summary of the paper through the essential elements such as, importance, aims, methods, results and conclusion. It is suggested that the Keywords may be arranged alphabetically with addition of the term "think aloud".
2. The introduction is well-composed, and adequately introduces the topic with a demonstration of the shortcomings of previous research. However, the researchers may add some ‘think aloud’ studies.
3. The literature review is adequately wide and deep with many up-to-date and main resources. Authors are advised to work more about the sections "reading strategies" and it may be subdivided into reading comprehension, think aloud, etc. or the authors should identify the correlation between such elements.
4. In the research questions, authors are recommended to add the adjective "Saudi" to identify the EFL learners involved.
5. The methods section is organized with discussion on the research design, population, selection of the participants, etc. The study identifies how the participants were chosen, the ethical consideration and the procedures followed as well as the instrument. Generally, the method used here can be replicated and reapplied.
6. Results were clearly presented and organized according to the two research questions. The discussion section interprets and compares the findings with previous research findings. However, there are minor mistakes in the use of in-text citations. Authors are advised to edit them, for example, "The study by Hoang Nguyen and Daniel R. Terry (2017); Nunaki, Damopolii, Kandowangko, and Nusantri (2019)".
7. The study ends with a pertinent conclusion and some recommendations, implementations and limitations. Suggestions for further research are also set.
8. The references are up-to-date and written according to the journal outline.

Is the work clearly and accurately presented and does it cite the current literature?

Yes

If applicable, is the statistical analysis and its interpretation appropriate?

Yes

Are all the source data underlying the results available to ensure full reproducibility?

Yes

Is the study design appropriate and is the work technically sound?

Yes

Are the conclusions drawn adequately supported by the results?

Yes

Are sufficient details of methods and analysis provided to allow replication by others?

Yes

Reviewer Expertise:

Applied Linguistics, corpus linguistics, English language

I confirm that I have read this submission and believe that I have an appropriate level of expertise to confirm that it is of an acceptable scientific standard, however I have significant reservations, as outlined above.

# Peer review: Reviewer response for version 1

**DOI:** 10.5256/f1000research.135163.r150900

**Published:** 2022-09-26

**Authors:** Asmara Shafqat

**Affiliations:** 1 Department of English, Humanities, NED University of Engineering and Technology, Karachi, Pakistan

The manuscript,  " Incorporating reading strategies for EFL undergraduate learners in Saudi Arabia: A think-aloud study" is a wonderful work in Saudi Arabia context. The researcher tried to put all the convincing ideas in a systematic organization of the research pattern, however, the article requires proofreading. There are issues of subject verb agreement at some places.

 Besides this, writing results should be according to the APA or other standardized writing style suggested by the journal's guidelines, for instance, mentioning P- value should be formally written and other values in the results.

 There is a suggestion about the number of the participants taken for the interview. It has been mentioned that there were two male interviewees and 1 female interviewee. There should be equal representation of the gender as it was done for the reading test and overall population of the study.

 There were 13 males and 13 females, therefore, at least there should be 2 female interviewees like there were 2 males for interview.

 Another noticeable thing is, there was nothing mentioned in the method which inferential statistical measure or researcher applied and on what parameters that statistical test was chosen to test the second research question.

 With the minor amendments, this manuscript can be accepted for indexing.

Is the work clearly and accurately presented and does it cite the current literature?

Partly

If applicable, is the statistical analysis and its interpretation appropriate?

Partly

Are all the source data underlying the results available to ensure full reproducibility?

Yes

Is the study design appropriate and is the work technically sound?

Yes

Are the conclusions drawn adequately supported by the results?

Yes

Are sufficient details of methods and analysis provided to allow replication by others?

Yes

Reviewer Expertise:

Academic reading and writing, Corpus linguistics, Pragmatics, Discourse, Corpus Driven Teaching Methods, Learning styles of the learners and teachers. EAP and ESP and all ELT issues.

I confirm that I have read this submission and believe that I have an appropriate level of expertise to confirm that it is of an acceptable scientific standard, however I have significant reservations, as outlined above.
